# Behavioral Evolutionary Analysis between the Government and Uncertified Recycler in China’s E-Waste Recycling Management

**DOI:** 10.3390/ijerph17197221

**Published:** 2020-10-02

**Authors:** Qixiang Wang, Linghui Kong, Jin Li, Bangyi Li, Fan Wang

**Affiliations:** 1 College of Economics and Management, Nanjing University of Aeronautics and Astronautics, Nanjing 210016, China; 270409@nau.edu.cn (Q.W.); wangzhe90@nuaa.edu.cn (B.L.); 2 School of Accounting, Guangdong University of Finance and Economics, Guangzhou 510320, China; 3 School of Management and E-Business, Key Research Institute-Modern Business Research Center, Zhejiang Gongshang University, Hangzhou 310018, China; jinli@mail.zjgsu.edu.cn; 4 School of Accounting, Zhejiang Gongshang University, Hangzhou 310018, China; wangfan1031@mail.zjgsu.edu.cn

**Keywords:** e-waste, uncertified recycler, government governance, evolutionary game

## Abstract

In many developing countries, the existence of the uncertified recycler seriously hinders the healthy development of the waste electrical and electronic equipment (WEEE or e-waste) recycling industry. As a result, how the government can regulate the uncertified recycler to improve environment and public health during the recycling processes has become a critical issue. To help tackle this issue, we build an evolutionary game model to study the interactions between the government and the uncertified recycler. We conduct stability analysis of each participant and obtain four asymptotically stable states. Furthermore, we conduct numerical simulations for comparative analysis based on the current situation of the Chinese e-waste recycling industry. Our results are as follows. First, there exist multiple asymptotically stable states for the government and the uncertified recycler, namely (no-governance, maintaining status quo), (governance, maintaining status quo), (governance, industrial upgrading), and (no-governance, industrial upgrading). Then, we verify the validity of the evolutionary game model through numerical simulations and find that penalty, supervision cost, additional investment cost, and financial subsidy can significantly influence the behavioral strategy of the government and the uncertified recycler. Finally, we find that the government should adopt the reward-penalty-supervision mechanism to promote the healthy development of the e-waste recycling industry and protect the environment and public health. Specifically, first, the government’s subsidy for the uncertified recycler has upper and lower limits. Exceeding the upper limit will result in an excessive financial burden to the government, while falling below the lower limit will hinder the uncertified recycler from technology upgrading. Second, the government should strengthen the supervision of the uncertified recycler and increase the punishment for violations. Third, the government should focus on controlling the supervision cost. Fourth, according to the asymptotically stable state (no-governance, industrial upgrading), the government should prepare to withdraw from the market when the uncertified recycler chooses industrial upgrading.

## 1. Introduction

The e-waste stream has become one of the fastest growing waste streams in the world and also one of the largest sources of heavy metals and organic pollutants in municipal wastes [1]. According to [2], the worldwide amount of e-wastes is estimated at 50 million tons in 2018, and the annual growth rate of e-wastes is expected to be 3–5%. In recent years, trans-border movement of e-wastes, which refers to illegal exportation to developing countries is quite common. A huge amount of e-wastes is exported to Asian countries such as China, India, and Pakistan and to African countries such as Ghana and Nigeria. Moreover, between 50% and 80% of e-wastes collected from developed countries is being exported each year [3]. This aggravates the situation of e-waste recycling in developing countries.

China, as the largest developing country in the world, was expected to produce 15.5 million tonnes of e-waste in 2020 [4]. China has established a relatively comprehensive e-waste recycling system and has approved a number of national certified recyclers (e.g., Qingdao Haier, Hangzhou Dadi, Beijing Huaxing, and Tianjin Datong) in e-waste recycling [5]. However, these certified recyclers, who are authorized by the government, are facing competitive pressures from uncertified recyclers, including private factories, collection points, and small workshops. When dealing with e-wastes, the uncertified recycler may treat the valuable parts by simply taking them apart, while the worthless parts are disposed by firing, dumping, and so on. This results in not only a waste of resources but also serious pollution to the environment [6]. However, as uncertified recyclers may offer a higher recovery price, they will receive the majority of the e-wastes. The uncertified recycler gradually grows with the development of local economy and relies on recycling secondary materials to make profit. At present, uncertified recycling channels are the main ways of recycling e-wastes in China [7]. Taking mobile phones for example, China’s mobile-cellular subscription volume reached 1.32 billion units by 2017 [8]. Furthermore, there were about 1 billion used mobile phones in China that needed to be handled in 2017, but the recycling rate of the certified recycler was less than 3%. What is more concerning is that uncertified recycling has become a major source of toxic heavy metals [9]. Heavy metals can reduce air quality and cause environmental pollution [10], and they also can be absorbed by humans [11]. Therefore, the uncertified recycler can not only cause serious pollution to the environment but also threaten public health and safety if their recycling behaviors are without legal permissions, toxicity preventions, and health protections [12]. However, uncertified recyclers can alleviate unemployment and promote social stability, so the government does not completely outlaw them [13].

In order to reduce the negative influence on the environment and public health of uncertified recycling, the Chinese government has adopted regulations on the activities of the uncertified recycler, such as Administrative Measures for Eligibility License for Disposal of Waste and Discarded Electrical and Electronic Products, and Regulation on the Administration of the Recovery and Disposal of Waste Electrical and Electronic products (2019 revised) [4,14]. These policies aim to guide the uncertified recycler to become certified and adopt treatment technologies that meet various requirements. Therefore, the first research objective of this paper is to study the impacts of government policies on the behavioral evolution of the uncertified recycler.

Although China has established a relatively comprehensive system for e-waste management, its implementation may have been ineffective [15]. Specifically, sometimes the government engages in lax enforcement and sometimes the rewards and punishments are inappropriate [16]. Moreover, the uncertified recycler may simply omit related laws. Therefore, the second objective of this paper is to study the interactions between the government and the uncertified recycler.

To achieve the two research objectives mentioned above, in this paper, evolutionary game theory is adopted. This theory explains the phenomenon of learning, competing, and adapting in the process of system evolution [17].Its most prominent feature is the assumption that each participant is boundedly rational in the evolution process. So far, this theory has been widely employed in fields such as supply chain management [18], computer science [19], and coal-mine safety [20]. By applying evolutionary game theory, we can obtain the payoff matrix and the replicator dynamic equation and then analyze the potential equilibrium points and asymptotically stable states. Obviously, the development of the e-waste recycling industry is a dynamic process, in which the government and the uncertified recycler are boundedly rational. In addition, the participants will choose different strategies based on the changing costs and benefits. Therefore, evolutionary game theory is appropriate to achieve our research objectives. 

The study emphasizes the following three research questions: (1) What is the payoff matrix of the bilateral participants in the evolutionary game model? (2) What are the equilibrium points and asymptotically stable states of the game participants? What are the required conditions to achieve each asymptotically stable state? (3) How do the main parameters in the model affect the behavioral evolution of the participants? 

In order to answer the questions above, we construct a bilateral evolutionary game model consisting of the government and the uncertified recycler. After conducting stability analysis of each participant, we obtain the asymptotically stable states of the e-waste recycling system by Jacobian matrix. Then, we verify the validity of the model and discuss how the main parameters impact the behavioral evolution trends of both participants.

The rest of this paper is organized as follows. Section 2 provides an overview of relevant research literature and emphasizes the research contributions of this paper. Section 3 builds the evolutionary game model consisting of the government and the uncertified recycler and analyzes the asymptotically stable states of the two participants. Section 4 conducts numerical simulations of the evolutionary game model based on the current situation of the Chinese e-waste recycling industry. Finally, Section 5 summarizes the findings of this paper and puts forward the corresponding policy recommendations.

## 2. Literature Review

The literature related to this paper focuses on three aspects: the first aspect is the management mechanism of the government in e-waste recycling industry, the second aspect is uncertified recyclers’ current state and role, and the third aspect is the interactions between the government and uncertified recyclers.

The government can employ a series of policies to regulate the e-waste recycling industry. These policies include subsidy, penalty, and mandated recovery target. By analyzing 109 Chinese e-waste recycling enterprises, Wang et al. [12] found that more flexible recycling product catalogs and funding standards are beneficial. In the meanwhile, the government providing targeted financial support can help develop the scale of the e-waste recycling industry and improve its standardization. Zhu et al. [16] investigated the effects of remanufactured product sale subsidies and remanufactured product donation subsidies on e-waste management and their economic, environmental, and social implications. Their results showed that a manufacturer receiving sales subsidies from the government can achieve better economic and environmental performance when the subsidy is low. Moreover, donation subsidies can offer the same benefit when the subsidy is high. Liu et al. [21] assumed that the manufacturer has the recycling qualification and analyzed the influence of fund policy on its recycling decision using a procedural model. Their results showed that applying for the recycling qualification is beneficial to the manufacturer. Moreover, with the increase of the financial subsidies and the reduction of the disposal fees, the profit of the manufacturer will improve. However, excessive financial subsidies or disposal fees may be detrimental to the environment. Wang et al. [22] discussed the role of the government’s punishment mechanism in the e-waste recycling industry by constructing a two-period, closed-loop supply chain model. Their results showed that the government’s punishment mechanism can not only reduce the disposal fee but also improve the e-waste recycling rate. Moreover, it can improve the profits of the recyclers and increase their willingness to remanufacture. Atasu et al. [23] focused on the influences of mandated targets on the e-waste management and explored the fairness of these targets. They found that an effective policy should make producers responsible for their own wastes and support green product design. However, the studies reviewed above focused on the effects of the government’s different policies on e-waste recycling without considering different recycling participants such as uncertified recyclers. In contrast, in this paper, we focus on the impact of government policies on uncertified recyclers.

Uncertified recyclers are sometimes called informal recyclers [24], unqualified recyclers [14], and irregular recyclers [25] in the literature. Briassoulis [26] and Guha-Khasnobis et al. [27] stated that uncertified means the sector is not within the scope of official governance and not approved and authorized by the government. Wilson et al. [28] found that in developing countries such as Philippines, India, Pakistan, and China, the proportion of uncertified recyclers is very high and their recycling rates are as high as 20–50%. Based on the current state of the e-waste recycling industry, the Chinese government has established a series of recycling pilot programs and supported many certified recyclers to implement waste management. However, these certified recyclers are less competitive compared with uncertified ones [29]. Gu et al. [30] conducted an empirical analysis on the stability and profitability of the uncertified recycler. They found that price and convenience incentivize consumers to sell used products to the uncertified recycler, who can buy used products from consumers at lower prices but still make profit due to its lower recycling costs [31]. In contrast, since the total cost of processing used products may exceed the total benefit from recycling the materials and components, the certified recycler may lose money [32]. The existence of uncertified recyclers is of great significance to the economic development of many developing countries. Hence, outlawing them can affect the stability of the e-waste recycling industry [33]. Wilson et al. [28,33] argued that the government should recognize the role of uncertified recyclers and integrate them into the waste management industry. Ardi and Leisten [34] employed the system dynamic method to simulate a variety of uncertified operations in e-waste management systems and they assessed the role of the uncertified recycler. At present, outlawing uncertified recyclers is an ineffective way in developing countries. For the government, the key is how to provide incentives to upgrade uncertified recyclers to certified ones [35], which will generate significant economic and social benefits [36]. In contrast, the government’s lax supervision of uncertified recyclers may hinder their sustainable development [37]. Most research on uncertified recyclers relies on qualitative research methods to analyze their behavior from a static perspective. Hence, there is a lack of dynamic and quantitative analysis. To fill this research gap, this paper uses evolutionary game theory to consider the positive and negative impacts of the uncertified recycler from a dynamic perspective and quantitatively analyzes its behavioral evolution.

So far, most research has only focused on the interaction between the government and the certified recycler. Esenduran et al. [38] and Toyasaki et al. [39] discussed the impacts of government regulations on the certified recycler’s two product take-back compliance schemes. They derived the required conditions to choose each of the two schemes. Chang et al. [40] studied a system composed of the government, a manufacturer, and a certified recycler. They showed that joint taxes and subsidies can improve recycling. However, these studies omitted the existence of uncertified recyclers. As far as we know, there is little research on the interaction between the government and uncertified recyclers from the perspective of operations management. Liu et al. [3] considered a dual channel with the certified recycler and the uncertified recycler and explored the impacts of the government’s subsidy on the certified recycler in the dual-channel competition. Li et al. [24] considered consumer’s different preference for certified and uncertified recyclers. They established three governance mechanisms to manage the uncertified collection channel. Zhou et al. [41] considered the self-financing subsidy scheme in China and constructed a two-period model consisting of a manufacturer and two recyclers to study the efficiency of Extended Producer Responsibility (EPR)_legislations. They find that higher subsidies might be detrimental to the manufacturer and the recycler. In this paper, we study the evolutionary behavior between the government and the uncertified recycler under the reward–penalty–supervision joint mechanism from a dynamic perspective. For clarity, we summarize the representative existing studies in Table 1.

In short, our paper differs from the existing studies in the literature and makes the following contributions:

(1) Different from the literature on certified or uncertified recyclers from a supply chain perspective, this paper analyzes the interaction and the evolution process between the government and the uncertified recycler from an evolutionary perspective. Thus, this research provides a new perspective to study the e-waste recycling industry.
(2) Different from the literature on uncertified recyclers assuming complete rationality, this paper explicitly studies the behavioral strategy of the uncertified recycler during the evolution of the e-waste recycling industry assuming boundedly rational participants. Our research provides a justification for the existence of the uncertified recycler.
(3) This paper proposes a reward–penalty–supervision mechanism for the government and analyzes its impact on the upgrading of the uncertified recycler. It also offers policy recommendations for the government to regulate uncertified recyclers for the development of the e-waste recycling industry.

## 3. Evolutionary Game Model

This paper takes the government and the uncertified recycler as the research subjects and analyzes their dynamic strategies according to the changes in the environment.

As the executor of e-waste recycling, the uncertified recycler has two development strategies, namely, industrial upgrading and maintaining status quo. Industrial upgrading means that its disposal activities after recycling satisfy the requirements of environmental laws by upgrading disposal technologies and equipment. This can protect the environment and public health by reducing or eliminating pollution. Maintaining status quo means that the uncertified recycler continues to rely on existing technologies and equipment to carry out illegal disposal activities of e-wastes.

As the regulator, the government has two management strategies for the e-waste recycling industry, namely, governance and no-governance [14,42]. With governance, the government can actively supervise the uncertified recycler’s behaviors of disposal and recycling and formulate policies to incentivize its industrial upgrading. Without governance, the government provides no supervision over the uncertified recycler.

### 3.1. Basic Assumptions and Model Building

This paper makes the following assumptions.

**Assumption 1.** Under the strategy of maintaining status quo, the uncertified recycler’s collection cost is denoted by $C_{1}$, the recycling cost is denoted by $C_{2}$, and the recycling benefit is denoted by $P_{1}$. Because the uncertified recycler uses relatively primitive techniques such as acid leaching, incineration, and landfill to deal with e-wastes, it will pollute the environment and hurt human health. Therefore, the government needs to pay a social and environmental cost E. Under the strategy of industrial upgrading, the uncertified recycler’s disposal activities conform to environmental laws by upgrading disposal technologies and equipment. Consequently, industrial upgrading can reduce environmental pollution and improve human health, thereby bringing a social and environmental benefit $R_{1}$ for the government. However, industrial upgrading requires additional investment cost $C_{3}$. After industrial upgrading, the recycling benefit of the recycler is denoted by $P_{2}$.

**Assumption 2.** With governance, the government actively supervises the uncertified recycler’s disposal and recycling and checks whether its behaviors satisfy the environmental standards. As a result, the government needs to pay supervision cost $C_{g}$ and obtain positive social benefit $R_{2}$. If the recycler chooses the industrial upgrading strategy, the government will provide subsidy, which is denoted by S. If the recycler chooses the strategy of maintaining the status quo and pollutes the environment, the government imposes the environmental tax F.

**Assumption 3.** The government chooses between the governance and no-governance strategy with a probability of x and 1−x, respectively. The uncertified recycler selects the strategy of industrial upgrading and maintaining status quo with a probability of y and 1−y, respectively.

Based on the assumptions above, the payoff matrix of the government and the uncertified recycler is shown in Table 2 where $P_{G}$ represents the revenue of the government and $P_{R}$ represents the revenue of the uncertified recycler.

### 3.2. Stability Analysis of the Government and the Uncertified Recycler

The government and the uncertified recycler are boundedly rational in the evolutionary game, and they can adjust their strategies to the changes in the environment. In this subsection, we use the replicator dynamic principle to calculate the expected revenue according to the payoff matrix of the evolutionary game [43,44,45]. Then, we obtain the replicator dynamic equations of each participant based on which stability analysis is conducted.

For the government, the expected revenue of choosing the governance strategy $E_{11}$, the expected revenue of choosing the no-governance strategy $E_{12}$, and the average expected revenue $E_{1}$ are respectively expressed as:

(1) $$\begin{array}{l} E_{11}=y(R_{1}+R_{2}-S-C_{g})+(1-y)(R_{2}+F-C_{g}-E)\\ \;=y(R_{1}+E-S-F)+R_{2}+F-C_{g}-E \end{array}$$

(2) $$\begin{array}{l} E_{12}=yR_{1}+(1-y)(-E)\\ \;=y(R_{1}+E)-E \end{array}$$

(3) $$E_{1}=xE_{11}+(1-x)E_{12}$$

Based on (1)–(3), the replicator dynamic equation of the government choosing the governance strategy can be written as:

(4) $$\begin{array}{l} dx/dt=x(E_{11}-E_{1})=x(1-x)(E_{11}-E_{12})\\ \;=x(1-x)[y(-S-F)+R_{2}+F-C_{g}] \end{array}$$

According to (4), when $y=\frac{R_{2}+F-C_{g}}{S+F}$, then dx/dt≡0, indicating the boundary of the stable state. If $y\neq \frac{R_{2}+F-C_{g}}{S+F}$, then x=0 and x=1, which are two stable points. Further, if $(dx/dt{)}^{\prime }<0$, then the equilibrium state represented by x is the asymptotically stable state of the system. Therefore, we take the derivative with respect to dx/dt: $(dx/dt{)}^{\prime }=(1-2x)[y(-S-F)+R_{2}+F-C_{g}]$. If $y>\frac{R_{2}+F-C_{g}}{S+F}$, then $(dx/dt{)}^{\prime }\left|{}_{x=0}\right.>0$ and $(dx/dt{)}^{\prime }\left|{}_{x=1}\right.<0$. At this time, x=1 is the stable equilibrium point and the government choosing the governance strategy is the stability strategy. On the contrary, if $y<\frac{R_{2}+F-C_{g}}{S+F}$, then $(dx/dt{)}^{\prime }\left|{}_{x=0}\right.<0$ and $(dx/dt{)}^{\prime }\left|{}_{x=1}\right.>0$. At this time, x=0 is the stable equilibrium point and the government choosing the no-governance strategy is the stability strategy.

For the uncertified recycler, the expected revenue of choosing the industrial upgrading strategy $E_{21}$, the expected revenue of choosing maintaining status quo strategy $E_{22}$, and the average expected revenue $E_{2}$ are respectively expressed as:

(5) $$\begin{array}{l} E_{21}=x(P_{2}+S-C_{1}-C_{2}-C_{3})+(1-x)(P_{2}-C_{1}-C_{2}-C_{3})\\ \;=xS+P_{2}-C_{1}-C_{2}-C_{3} \end{array}$$

(6) $$\begin{array}{l} E_{22}=x(P_{1}-F-C_{1}-C_{2})+(1-x)(P_{1}-C_{1}-C_{2})\\ =x(-F)+P_{1}-C_{1}-C_{2} \end{array}$$

(7) $$E_{2}=yE_{21}+(1-y)E_{22}$$

Based on (5)–(7), the replicator dynamic equation of the uncertified recycler choosing the industrial upgrading strategy can be written as:

(8) $$\begin{array}{l} dy/dt=y(E_{21}-E_{2})=y(1-y)(E_{21}-E_{22})\\ \;=y(1-y)[x(S+F)+P_{2}-C_{3}-P_{1}] \end{array}$$

According to (8), if $x=\frac{P_{1}+C_{3}-P_{2}}{S+F}$, then dy/dt≡0, indicating the boundary of the stable state. If $x\neq \frac{P_{1}+C_{3}-P_{2}}{S+F}$, then y=0 and y=1 are the two stable points. Further, if $(dy/dt{)}^{\prime }<0$, then the equilibrium state represented by y is the asymptotically stable state of the system. We take the derivative with respect to dy/dt: $(dy/dt{)}^{\prime }=(1-2y)[x(S+F)+P_{2}-C_{3}-P_{1}]$. If $x>\frac{P_{1}+C_{3}-P_{2}}{S+F}$, then $(dy/dt{)}^{\prime }\left|{}_{y=0}\right.>0$ and $(dy/dt{)}^{\prime }\left|{}_{y=1}\right.<0$. At this time, y=1 is the stable equilibrium point and the uncertified recycler choosing industrial upgrading strategy is the stability strategy. On the contrary, if $x<\frac{P_{1}+C_{3}-P_{2}}{S+F}$, then $(dy/dt{)}^{\prime }\left|{}_{y=0}\right.<0$ and $(dy/dt{)}^{\prime }\left|{}_{y=1}\right.>0$. At this time, y=0 is the stable equilibrium point and the uncertified recycler choosing maintaining status quo strategy is the stability strategy.

### 3.3. Solutions of the Asymptotically Stable States

Equations (4) and (8) above constitute the two-dimensional, dynamic system of the evolutionary game model:

(9) $$\left\{\begin{array}{l} dx/dt=x(1-x)[y(-S-F)+R_{2}+F-C_{g}]\\ dy/dt=y(1-y)[x(S+F)+P_{2}-C_{3}-P_{1}] \end{array}\right.$$

Further, let $\left\{\begin{array}{l} dx/dt=0\\ dy/dt=0 \end{array}\right.$, then we can obtain four pure strategy equilibrium points of the system, which are (0,0),(0,1),(1,0), and (1,1), respectively. These four equilibrium points constitute the boundary of the solutions of the evolutionary game model, which is $\left\{(x,y)\left|0\leq x,y\leq \left.1\right\}\right.\right.$. In addition, there exists a mixed strategy equilibrium point $\left(x^{*},y^{*}\right)$, which satisfies $0\leq x^{*},y^{*}\leq 1$ where

(10) $$x^{*}=\frac{P_{1}+C_{3}-P_{2}}{S+F}.y^{*}=\frac{R_{2}+F-C_{g}}{S+F}$$

Whether the five equilibrium points (including four pure strategy equilibrium points and one mixed strategy equilibrium point) become the asymptotically stable states needs to be determined by using the Jacobian matrix [17]. The Jacobian matrix of our evolutionary game model is as follows:

(11) $$J=\left[\begin{array}{cc} \partial (dx/dt)/\partial x & \partial (dx/dt)/\partial y\\ \partial (dy/dt)/\partial x & \partial (dy/dt)/\partial y \end{array}\right]=\left[\begin{array}{cc} J_{11} & J_{12}\\ J_{21} & J_{22} \end{array}\right]$$

where $J_{11}=(1-2x)[y(-S-F)+R_{2}+F-C_{g}]$, $J_{12}=x(1-x)(-S-F)$, $J_{21}=y(1-y)(S+F)$, and $J_{22}=(1-2y)[x(S+F)+P_{2}-C_{3}-P_{1}]$.

It is known that $\det J=J_{11}J_{22}-J_{12}J_{21}$, $trJ=J_{11}+J_{22}$. The criterions for judging the local stability of the equilibrium points by using Jacobian matrix are as follows. If detJ>0 and trJ<0, then the equilibrium point is the asymptotically stable state of the system. If detJ>0 and trJ>0, then the equilibrium point is the instability point of the system. If trJ=0, then the equilibrium point is the saddle point. Therefore, we calculate the determinants and the traces of each equilibrium point, and then judge their plus or minus characteristics to determine asymptotically stable states of the system. The determinants and the traces of each equilibrium point are shown in Table 3.

In Table 3, $\lambda^{*}=\frac{\left(P_{L}+\Delta C-P_{H})(S+T+P_{H}-P_{L}-\Delta C)(G_{2}+T-C_{g})(S+C_{g}-G_{2}\right)}{{\left(S+T\right)}^{2}}$.

As can be seen from Table 3, the mixed strategy equilibrium point $\left(x^{*},y^{*}\right)$ is the saddle point of the system. Next, we will show why the saddle point cannot be an asymptotically stable state. 

**Figure 1:**
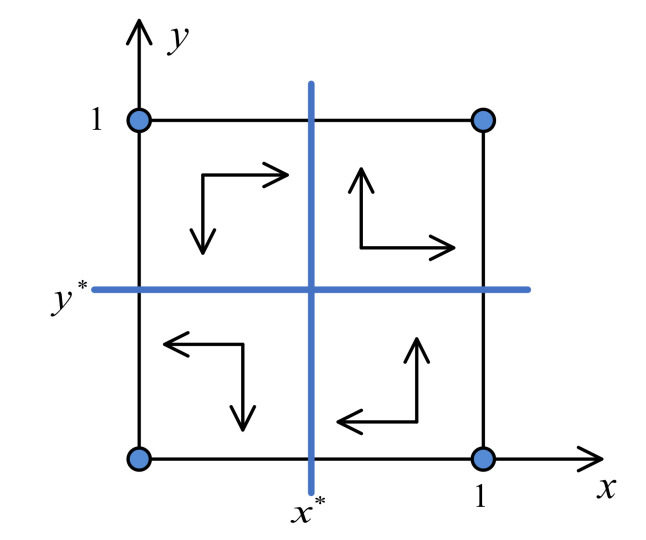
Different locations of the saddle point $\left(x^{*},y^{*}\right)$.

The change of its location determines which asymptotically stable state the system eventually evolves to, as shown in Figure 1. In the first quadrant of Figure 1, the location of the saddle point is $\left(x^{*}>0.5,y^{*}>0.5\right)$. Here, the asymptotically stable state of the system is (1,1), that is, the government chooses the governance strategy and the uncertified recycler chooses the industrial upgrading strategy. In the second quadrant, the location of the saddle point is $\left(x^{*}<0.5,y^{*}>0.5\right)$. Here, the asymptotically stable state of the system is (1,0), that is, the government chooses the governance strategy and the uncertified recycler chooses the maintaining status quo strategy. In the third quadrant, the location of the saddle point is $\left(x^{*}<0.5,y^{*}<0.5\right)$. Here, the asymptotically stable state of the system is (0,0), that is, the government chooses the no-governance strategy and the uncertified recycler chooses the maintaining status quo strategy. In the fourth quadrant, the location of the saddle point is $\left(x^{*}>0.5,y^{*}<0.5\right)$. Here, the asymptotically stable state of the system is (0,1), that is, the government chooses the no-governance strategy and the uncertified recycler chooses the industrial upgrading strategy. Based on the analysis above, the saddle point’s different locations correspond to different asymptotically stable states, so it cannot be an asymptotically stable state.

Therefore, the remaining four equilibrium points can become asymptotically stable states of the system under different conditions (see Table 4).

**Figure 2:**
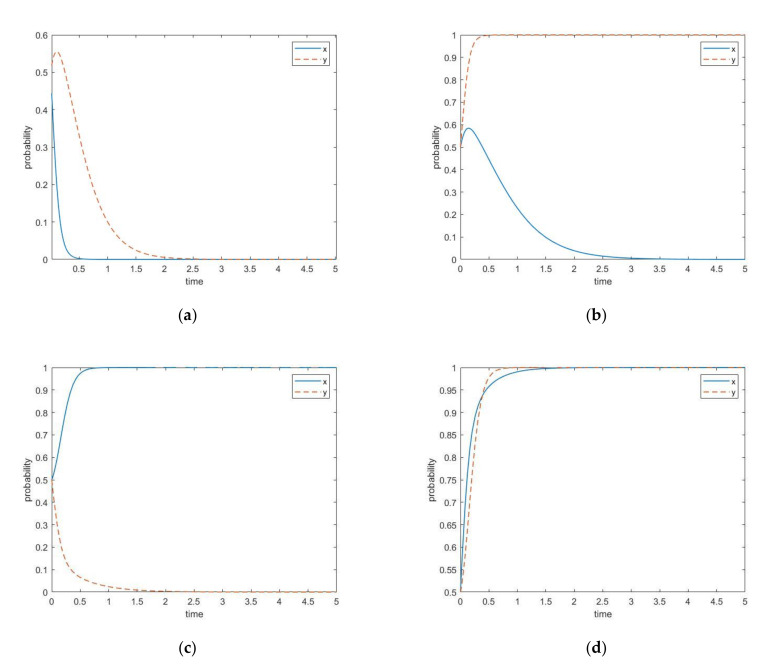
The evolutionary paths of the four asymptotically stable states. (**a**) Asymptotically stable state (0,0); (**b**) asymptotically stable state (0,1); (**c**) asymptotically stable state (1,0); (**d**) asymptotically stable state (1,1).

Table 4 shows that the evolutionary game model has multiple asymptotically stable states. To achieve the asymptotically stable state (0,0), the behaviors of both participants should satisfy condition (1):$(R_{2}+F)<C_{g}$ and $(P_{2}-C_{3})<P_{1}$. That is, the government’s supervision cost is higher than the sum of the social benefit of governance and the penalty, and the revenue of the uncertified recycler choosing the strategy of maintaining status quo exceeds the net revenue of industrial upgrading. The evolutionary path is shown in Figure 2a. It can be seen from the figure that with the passage of time, the probability of the government choosing governance decreases rapidly, and finally its behavioral strategy evolves into no-governance. The probability of the uncertified recycler choosing industrial upgrading first increases slightly then decreases rapidly, and its strategy finally evolves to maintaining status quo.

To achieve the asymptotically stable state (0,1), the behaviors of both participants should satisfy condition (2): $R_{2}<(S+C_{g})$ and $(P_{2}-C_{3})>P_{1}$. That is, the sum of government’s supervision cost and the financial subsidy exceeds the social benefit of the government choosing the governance strategy, and the net benefit of the uncertified recycler choosing the industrial upgrading strategy exceeds the benefit from the strategy of maintaining status quo. The evolutionary path is shown in Figure 2b. It can be seen that the probability of government choosing the governance strategy first rises, then slowly decreases, and finally its behavioral strategy evolves into no-governance. The uncertified recycler reaches equilibrium at a rapidly and chooses the industrial upgrading strategy.

To achieve the asymptotically stable state (1,0), the behaviors of both participants should satisfy condition (3): $(R_{2}+F)>C_{g}$ and $\left(S+P_{2}-C_{3})<(P_{1}-F\right)$. That is, the sum of social benefit of governance and the penalty is higher than the government’s supervision cost, and the net benefit of the uncertified recycler from the industrial upgrading strategy is lower than the net benefit from choosing maintaining status quo strategy. The evolutionary path is shown in Figure 2c. It can be seen that with the passage of time, the government reaches equilibrium at an extremely fast rate and chooses the strategy of governance. The uncertified recycler reaches equilibrium at a relatively slowly and chooses the maintaining status quo strategy.

To achieve the asymptotically stable state (1,1), the behaviors of both participants should satisfy condition (4): $R_{2}>(S+C_{g})$ and $\left(S+P_{2}-C_{3})>(P_{1}-F\right)$. That is, the social benefit of the government’s governance is higher than the sum of supervision cost and financial subsidy, and the net benefit of the uncertified recycler choosing the industrial upgrading strategy exceeds the net benefit from choosing maintaining status quo strategy. The evolutionary path is shown in Figure 2d. It can be seen that with the passage of time, the government reaches equilibrium at a relatively fast rate and chooses the strategy of governance. The uncertified recycler reaches equilibrium at an extremely fast rate and chooses the strategy of industrial upgrading.

## 4. Numerical Simulation

In Section 3, we obtain five equilibrium points (including four pure strategies and one mixed strategy equilibrium solutions) of the system by solving the evolutionary game model. We further obtain its asymptotically stable states based on the Jacobian matrix. Based on the analysis of the four asymptotically stable states, it can be seen that different parameter values lead to different asymptotically stable states of the system. Therefore, we use MATLAB R2019a to investigate how the main parameters impact the evolutionary trends of the government and the uncertified recycler and reveal the internal evolutionary mechanism through numerical simulations.

**Figure 3:**
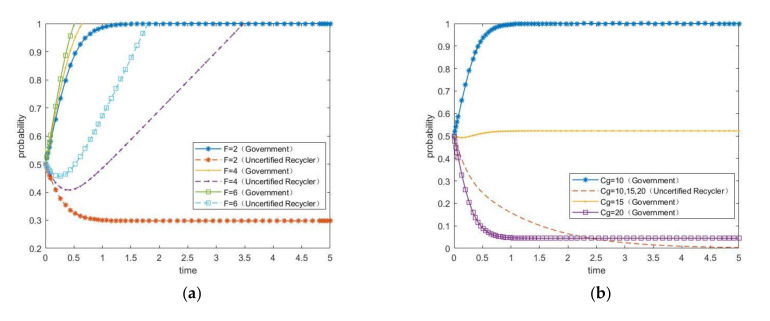

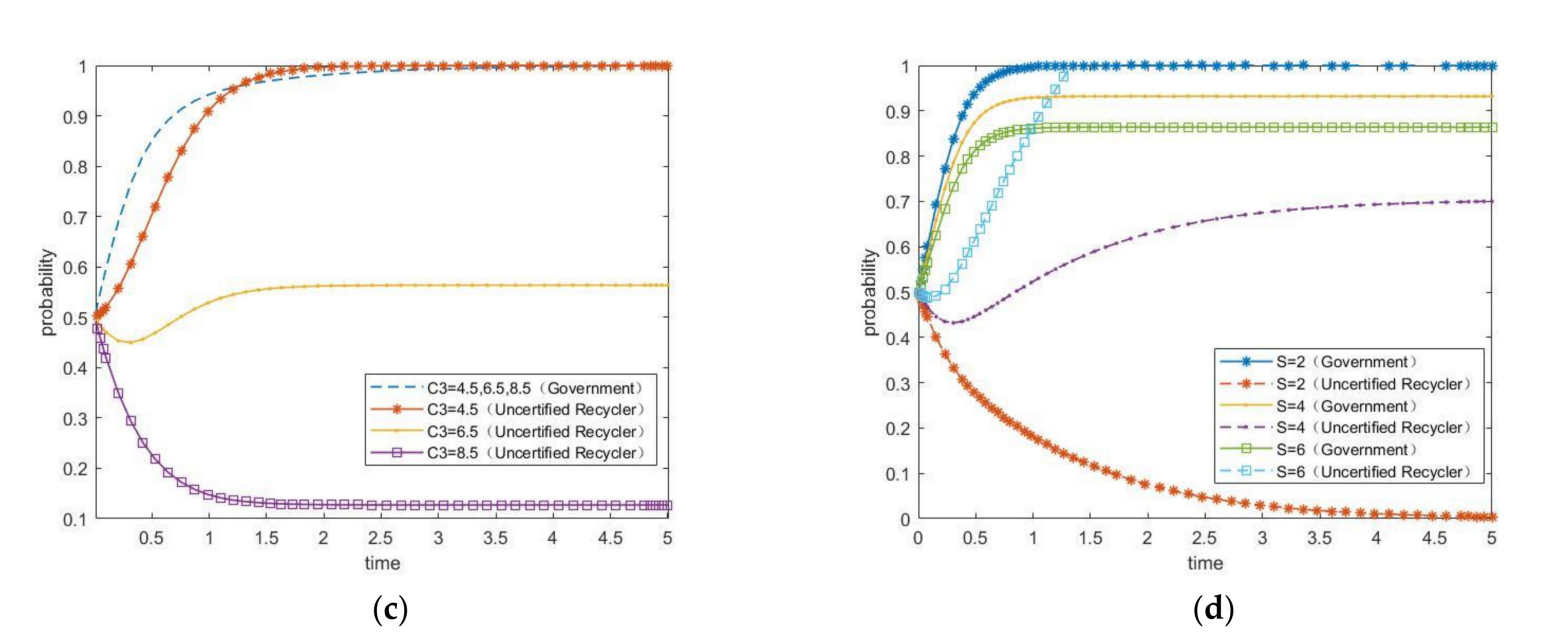
The impacts of the parameters on the evolution of the two participants’ behaviors. (**a**) Penalty; (**b**) supervision cost; (**c**) additional investment cost; (**d**) subsidy.

The current situation of the Chinese e-waste recycling industry is as follows. The government is fully aware of the importance of protecting the environment and public health. Hence, it will enforce strict supervision and governance on the uncertified recycler, who cannot obtain subsidies for recycling e-waste products. At the same time, the additional investment cost required to upgrade to the certified recycler is too high. Therefore, most uncertified recyclers will not choose industrial upgrading. This corresponds to the asymptotically stable state (1,0) in the evolutionary game model. Therefore, the parameter values should satisfy condition (3): $(R_{2}+F)>C_{g}$ and $\left(S+P_{2}-C_{3})<(P_{1}-F\right)$. To this end, they are set as follows: $R_{2}=15$,F=2,$C_{g}=10$,$P_{2}=5$,$C_{3}=8$, $P_{1}=3$, and S=3. Next, we conduct numerical simulations for the penalty F, supervision cost $C_{g}$, additional investment cost $C_{3}$, and financial subsidy S, respectively. The results are plotted Figure 3a–d, which shows the impacts of the penalty, the supervision cost, the additional investment cost, and the financial subsidy, respectively, on the strategic choices of the two participants. 

For Figure 3a, the values of F are set as 2, 4, and 6. It can be seen that with the improvement of the penalty, the speed of the government reaching stable equilibrium (choosing governance strategy) is increasingly faster. The probability of the uncertified recycler choosing industrial upgrading generally rises, and it is more sensitive to changes of the penalty. The reason for this evolutionary process is that the gradual increase in penalty brings higher government benefit, so the government tends to choose the governance strategy. For the uncertified recycler, a lower penalty means a lower cost to choose the maintaining status quo strategy, and the difference between the benefit of choosing the maintaining status quo strategy and the penalty is greater than the net benefit of choosing the industrial upgrading strategy. Thus, it ignores the penalty and chooses the maintaining status quo strategy. When the penalty continues to increase to be even higher than the benefit obtained from maintaining status quo, the uncertified recycler cannot ignore the penalty any more. Thus, the willingness of the uncertified recycler to choose the industrial upgrading strategy becomes very strong. 

For Figure 3b, the values of $C_{g}$ are set as 10, 15, and 20. We can see that with the increase in the supervision cost, the probability of the government choosing the governance strategy generally decreases. Because the excessive supervision cost increases its financial burden, the probability of the government choosing the governance strategy continues to decline. Moreover, it is more likely that the government will eventually choose the no-governance strategy. For the uncertified recycler, the change of the supervision cost has no impact on its choice of behavioral strategy.

For Figure 3c, the values of $C_{3}$ are set as 4.5, 6.5, and 8.5. We can see that with the continuous increase in the additional investment cost, the probability of the uncertified recycler choosing the industrial upgrading strategy declines overall. If it chooses industrial upgrading, the government’s financial subsidy is not enough to make up for the excessive additional investment cost. If it chooses maintaining status quo, the government’s penalty is far less than the additional investment cost. Therefore, if the additional investment cost is too high, the uncertified recycler eventually evolves into maintaining status quo. For the government, the change of the additional investment cost has no impact on its choice of behavioral strategy.

For Figure 3d, the values of S are set as 2, 4, and 6. It can be seen that with the increase in the subsidy, the probability of the government choosing the governance strategy decreases overall. The probability of the uncertified recycler choosing the industrial upgrading strategy increases overall, and it is more sensitive to the change of the subsidy. The reason for this evolutionary process may be that increased subsidy leads to increases government fiscal expenditure, which can reduce its enthusiasm to promote industrial upgrading. However, it still has the obligation to promote the sustainability, so its probability of choosing the governance strategy cannot decline too much. As can be seen from Figure 3d, its probability of choosing the governance strategy is greater than 0.8 under the three different subsidies. For the uncertified recycler, the increase in the subsidy can compensate for more cost expenditure. As a result, it chooses industrial upgrading to ease the penalty of the government, and its probability of choosing the industrial upgrading strategy would continue to increase.

## 5. Conclusions 

In order to better study the evolutionary mechanism of the government and the uncertified recycler in e-waste recycling industry, this paper constructs a two-party, evolutionary game model and analyzes their learning behavior and strategy adjustments in a long-term process. Our main conclusions are as follow. First, there are multiple asymptotically stable states in the evolutionary game model including (0,0), (0,1), (1,0), and (1,1). Second, higher penalties and subsidies can encourage the cooperation between the government and the uncertified recycler. The increase of the supervision cost will reduce the willingness of the government to choose the governance strategy. Likewise, the increase of the additional investment cost will reduce the willingness of the uncertified recycler to choose the industrial upgrading strategy.

Based on the research findings above, the following policy recommendations are proposed. First, the government should lead and make full use of the reward–penalty–supervision mechanism to promote the healthy development of the e-waste recycling industry. In the current Chinese e-waste recycling industry, uncertified recyclers typically prefer the strategy of maintaining status quo because its net benefit is higher than that from industrial upgrading. Thus, the government should increase financial subsidy for uncertified recyclers so that they would choose the industrial upgrading strategy. Moreover, excessive subsidies will burden the government financially and reduces its willingness to regulate the e-waste recycling industry. On the other hand, lower subsidies will reduce the willingness of the uncertified recycler to upgrade itself to a certified one. This will also hinder the healthy development of the e-waste recycling industry. Second, due to the weak punishment at this stage, the uncertified recycler may ignore it to maximize profit. Therefore, the government should strengthen the supervision of the uncertified recycler and increase the punishment for violations. If the penalty is too high, it will overburden some uncertified recyclers so that they would withdraw from the market. If the penalty is too low, the certified recycler may speculate and pursue illegal recycling. Third, the government should focus on controlling the supervision cost to reduce its financial burden. Specific supervision measures may include implementing multi-departmental joint supervision, following the principles of randomness (random checking the uncertified recycler) and transparency in the supervision process and diversifying the supervision subjects. Fourth, according to the asymptotically stable state (no-governance, industrial upgrading), the government should prepare to withdraw from the market with the aim of reducing intervention when uncertified recyclers choose industrial upgrading.

In this paper, we construct a two-party, evolutionary game model including the government and the uncertified recycler. However, consumers are also important participants in the development of the e-waste recycling industry. Therefore, future research can incorporate consumer behaviors to build a tripartite evolutionary game model. Second, our paper only considers whether or not the government should govern the uncertified recycler but omits the issue of governance intensity. Therefore, future research can conduct theoretic and empirical research to examine the role of the governance intensity in the e-waste recycling industry. Last but not least, the current research can be extended to the domains of supply chain management or the corporate social responsibility related to the recycling industry.

## Figures and Tables

**Table 1:** Summary of the representative existing studies.

Authors	The Management Mechanism of the Government			The Recycler		The Interactions Between Them		Completely Rational	Bounded Rationality
	Subsidy	Penalty	Mandated Target	Qualitative Analysis	Quantitative Analysis	Static Game	Dynamic Game		
Wang et al., 2018	√		√		√	√		√	
Zhu et al., 2017	√				√	√		√	
Liu et al., 2017	√				√	√		√	
Wang et al., 2018	√	√			√	√		√	
Atasu et al., 2009			√		√	√		√	
Briassoulis, 2016	√	√		√					
Guha-Khasnobis et al., 2006	√	√		√					
Wilson et al., 2009	√	√		√					
Hicks et al., 2005	√			√					
Gu et al., 2016	√				√	√		√	
Yang et al., 2008	√	√	√	√					
He et al., 2006	√	√		√					
Wilson et al., 2006	√	√		√					
Ardi et al., 2016	√	√			√		√	√	
Chi et al., 2011	√	√	√	√					
Perera et al., 1996					√				
Nzeadibe et al., 2009	√	√			√				
Esenduran et al., 2015	√				√	√		√	
Toyasaki et al., 2011	√				√	√		√	
Chang et al., 2019	√	√			√	√		√	
Liu et al., 2016	√				√	√		√	
Li et al., 2017	√	√			√	√		√	
Zhou et al., 2017	√				√	√		√	
This paper	√	√	√		√		√		√

**Table 2:** The payoff matrix in the evolutionary game.

		The Uncertified Recycler	
		Industrial Upgrading (y)	Maintaining Status Quo (1−y)
The government	Governance (x)	$P_{G}:R_{1}+R_{2}-S-C_{g}$; $P_{R}:P_{2}+S-C_{1}-C_{2}-C_{3}$	$P_{G}:R_{2}+F-C_{g}-E$; $P_{R}:P_{1}-F-C_{1}-C_{2}$
	No-governance (1−x)	$P_{G}:R_{1}$; $P_{R}:P_{2}-C_{1}-C_{2}-C_{3}$	$P_{G}:-E$; $P_{R}:P_{1}-C_{1}-C_{2}$

**Table 3:** The determinants and the traces of each equilibrium point.

Equilibrium Point	detJ	trJ
(0,0)	$\left(R_{2}+F-C_{g})(P_{2}-C_{3}-P_{1}\right)$	$\left(R_{2}+F-C_{g})+(P_{2}-C_{3}-P_{1}\right)$
(0,1)	$\left(R_{2}-S-C_{g})(P_{1}+C_{3}-P_{2}\right)$	$\left(R_{2}-S-C_{g})+(P_{1}+C_{3}-P_{2}\right)$
(1,0)	$\left(C_{g}-R_{2}-F)(S+F+P_{2}-C_{3}-P_{1}\right)$	$\left(C_{g}-R_{2}-F)+(S+F+P_{2}-C_{3}-P_{1}\right)$
(1,1)	$\left(R_{2}-S-C_{g})(S+F+P_{2}-C_{3}-P_{1}\right)$	$\left(C_{g}+S-R_{2})-(S+F+P_{2}-C_{3}-P_{1}\right)$
$\left(x^{*},y^{*}\right)$	$\lambda^{*}$	0

**Table 4:** The asymptotically stable states and their required conditions.

Asymptotically Stable States	Required Conditions
(0,0)	Condition (1): $(R_{2}+F)<C_{g}$,$(P_{2}-C_{3})<P_{1}$
(0,1)	Condition (2): $R_{2}<(S+C_{g})$,$(P_{2}-C_{3})>P_{1}$
(1,0)	Condition (3): $(R_{2}+F)>C_{g}$,$\left(S+P_{2}-C_{3})<(P_{1}-F\right)$
(1,1)	Condition (4): $R_{2}>(S+C_{g})$,$\left(S+P_{2}-C_{3})>(P_{1}-F\right)$
